# Sequence-Specific Detection of DNA Strands Using a Solid-State Nanopore Assisted by Microbeads

**DOI:** 10.3390/mi11121097

**Published:** 2020-12-11

**Authors:** Yin Zhang, Zengdao Gu, Jiabin Zhao, Liying Shao, Yajing Kan

**Affiliations:** Jiangsu Key Laboratory for Design and Manufacture of Micro-Nano Biomedical Instruments, School of Mechanical Engineering, Southeast University, Nanjing 211189, China; 220190300@seu.edu.cn (Z.G.); 220180274@seu.edu.cn (J.Z.); sly950904@163.com (L.S.); yajingkan@seu.edu.cn (Y.K.)

**Keywords:** nanopore, microbead, DNA detection

## Abstract

Simple, rapid, and low-cost detection of DNA with specific sequence is crucial for molecular diagnosis and therapy applications. In this research, the target DNA molecules are bonded to the streptavidin-coated microbeads, after hybridizing with biotinylated probes. A nanopore with a diameter significantly smaller than the microbeads is used to detect DNA molecules through the ionic pulse signals. Because the DNA molecules attached on the microbead should dissociate from the beads before completely passing through the pore, the signal duration time for the target DNA is two orders of magnitude longer than free DNA. Moreover, the high local concentration of target DNA molecules on the surface of microbeads leads to multiple DNA molecules translocating through the pore simultaneously, which generates pulse signals with amplitude much larger than single free DNA translocation events. Therefore, the DNA molecules with specific sequence can be easily identified by a nanopore sensor assisted by microbeads according to the ionic pulse signals.

## 1. Introduction

The sequence-specific detection of DNA or RNA targets plays an important role in molecular diagnosis and therapy applications [1,2,3,4]. The commonly used polymerase chain reaction (PCR)-based methodologies are considered as the “gold standards” for nucleic acid quantification [5,6]. However, time-consuming and expensive amplification steps of this technique limit its application in clinic. In recent years, some new PCR-free biosensors based on electrochemical [7,8,9,10,11] and optical [12,13,14,15] technologies, which are capable of sensing individual molecules, have been developed for bioanalysis and diagnosis [16]. One of the most effective and promising approach is nanopore based sensing due to its high throughput and low-cost performance [17,18]. 

In 2001, Howorka et al. [1] first identified the target ssDNA by an engineered protein nanopore sensor, which was built by attaching a ssDNA oligomer probe within the lumen of an α-HL pore. Gu’s group [19] used a programmable oligonucleotide probe to hybridize to the target miRNAs. Due to the limitation of the α-HL pore size, a probe-specific microRNA duplex passing through the pore will generate a signature multi-level pulse signal which is different from the signal induced by the probe or microRNA. Based on that methodology, peptide nucleic acid (PNA) [20,21,22] and locked nucleic acid (LNA) [23] probes were also applied in genetic biomarker detection for enhancing the sensitivity and selectivity. In addition, each kind of probe can be modified with a distinct barcode, so that multiple targets can be simultaneously distinguished by specific ionic signals [24]. However, protein nanopores still face the issues of limited membrane stability and difficulty in high throughput [25]. Instead, the solid-state nanopore exhibits the advantages of robustness, durability, modifiable surface property and tunable size. Dekker’s group [26] first utilized solid-state nanopores to distinguish single- and double-stranded nucleic acid molecules with their length longer than 10 kb. In 2010, Wanunu et al. [27] quantified the concentration of miRNA-probe hybrids by using a 3 nm pore in a 7 nm thick silicon nitride membrane. However, in their work, it requires an additional miRNA targets enrichment step before nanopore sensor detection. Meller and co-authors [28,29] bonded PNA probes to dsDNA and identified the specific internal sequences on dsDNA with sub-5 nm silicon nitride pores. Similarly, a PNA–polyethylene glycol (PEG) complex [30], Cas9 [31] and streptavidin-labelled sequence motif [32] were utilized to label target DNA and able to create additional blockade in ionic current. While those research studies often require the diameter of pores small enough to precisely identify the characteristic size change of target DNA hybridized with a probe. 

An alternative approach to detect target oligonucleotide is selectively binding the targets to probe-coated beads [33,34]. Ling’s group [35,36] used nanobeads to slow down the DNA translocation and detected DNA hybridizations. Booth et al. [37] demonstrated a variable pressure method to characterize the surface charge of nanobeads by nanopore resistive pulse sensing. In this way, they could discriminate between the ‘‘probe’’ and ‘‘target’’ bound beads. Schmidt’s group [38,39] proposed to sequence-specifically capture DNA by PNA probes conjugated beads, which leads to the neutral beads become negatively charged. Then the target attached beads were able to be electrically driven to the sensing zone of the nanopore and cause signature pulse signals. Additionally, PNA probes conjugated beads have shown promise for the detection of microRNA [40], rRNA [41], and circular DNA [42] targets with specific sequence. However, those detected pulse signals in most previous studies were generated by the beads rather than the single target oligonucleotide.

In this paper, the target DNA was hybridized with the biotinylated probe and then bonded to the streptavidin-coated microbeads. We directly detected the target DNA molecules attached on microbeads and free DNA molecules by using nanopores with diameters significantly smaller than the microbeads. In addition, we sought to investigate the effects of the microbeads on DNA transport behaviors and identified the target DNA molecules based on ionic pulse signals.

## 2. Materials and Methods 

**Figure 1:**
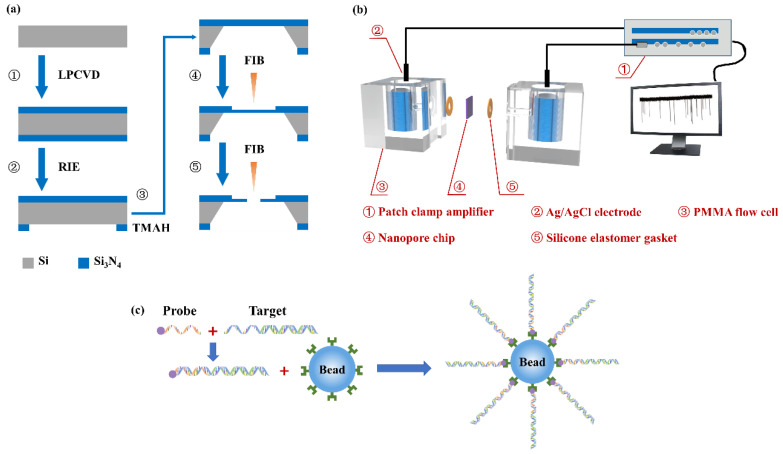
Schematic illustrations of (**a**) the nanopore fabrication process, (**b**) the nanopore-based detection setup and (**c**) the process of DNA selectively binding to the microbead.

The nanopore fabrication process is shown in Figure 1a. Two 100 nm thick low-stress silicon nitride membranes were deposited on both sides of the <100> silicon substrate by low pressure chemical vapor deposition (LPCVD). A 720 μm × 720 μm square release window was patterned on the backside of a silicon nitride membrane by photolithography and reactive ion etching (RIE). After that, the silicon substrate was etched using tetramethylammonium hydroxide (TMAH) solution with a mass fraction of 25% to expose a 160 μm × 160 μm, free-standing silicon nitride membrane. Then, a region with a size of 500 nm × 500 nm at the center of the silicon nitride membrane was milled by focused ion beams (FIB) to reduce the thickness to 20 nm. Nanopores were eventually drilled on the milled region by the FIB. 

A nanopore chip was treated in oxygen plasma for 30 s on both sides, and then assembled into a polymethylmethacrylate (PMMA) flow cell, as shown in Figure 1b. Two Ag/AgCl electrodes were connected to a patch clamp amplifier (Axon CNS 700B, Molecular Devices LLC, San Jose, CA, USA) and, respectively, inserted into two chambers of the flow cell, which were fulfilled with degassed and filtered 1 M KCl solution (buffered with 10 mM Tris-HCl and 1 mM Ethylene Diamine Tetraacetic Acid (EDTA) to pH 8). The DNA sample was added into the cis chamber, where it was electrically grounded. Positive or negative potentials were applied to the trans chamber. Current traces were measured at 250 kHz with a 10 kHz low-pass filter. All nanopore sensing experiments were taken inside a dark Faraday cage.

Streptavidin-coated polystyrene beads with a 1.5 μm diameter were obtained from Sigma-Aldrich (Shanghai, China). λ-DNAs (48.5 kbp) were purchased from Takara BIO Inc. (Shiga, Japan) DNA probes were purchased from Sangon Biotech (Shanghai, China) Co., Ltd. The probe with the sequence P-5’ -GGGCGGCGACCTT-3’ -B was phosphorylated (P) at the 5’ end and biotinylated (B) at the 3’ end. As shown in Figure 1c, the probe could be hybridized to the cos site of the λ-DNA using the standard hybridization protocol [43]. Finally, the DNA-probe complexes were mixed with streptavidin-coated polystyrene beads and incubated at room temperature for 3 h.

## 3. Results and Discussion

**Figure 2:**
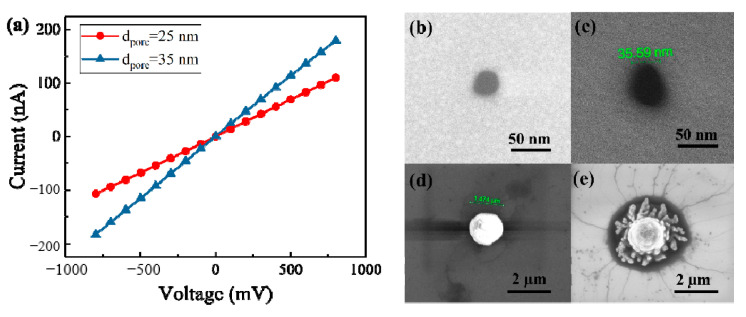
(**a**) Current–voltage curves for nanopores with diameters of 25 nm and 35 nm in 1M KCl solution; (**b**) and (**c**) SEM images of nanopores with diameters of approximately 26 nm and 36 nm; (**d**) SEM image of a polystyrene bead; (**e**) SEM image of a polystyrene bead after incubating with DNA–probe complexes.

We fabricated two nanopores in 20 nm thickness SiN membranes. Figure 2a presents the current–voltage curves for nanopores with 1 M KCl solution. The pore diameter *d*_pore_ could be calculated by [44]:

(1) $$G=\sigma {\left(\frac{4l}{\pi d_{\mathrm{pore}}^{2}}+\frac{1}{d_{\mathrm{pore}}}\right)}^{-1}$$

where *G* is the ionic conductance of a cylindrical nanopore, σ is the bulk electrolytic conductivity (for 1M KCl, σ = 11.18 S/m, at 25 °C), *l* refers to the length of a nanopore. The calculated nanopore diameters by Equation (1) are 25 nm and 35 nm, respectively. Figure 2b,c present the scanning electron microscope (SEM) images of these two nanpores. From the SEM images, diameters of nanopores were measured at approximately 26 nm and 36 nm. Considering measurement errors, we think measured diameter is consistent with the calculated value. 

In this paper, if probe could hybridize with target DNA molecules due to the matched sequence, the DNA–probe complexes would link to the microbeads through the streptavidin–biotin bond. Otherwise, the test sample is composed of microbeads with probes attached and the mismatched DNA molecules. Figure 2d,e are the SEM images of polystyrene beads before and after incubating with DNA–probe complexes. Filaments around the microbead shown in Figure 2e confirm that DNA–probe complexes have successfully bonded to the microbead. 

**Figure 3:**
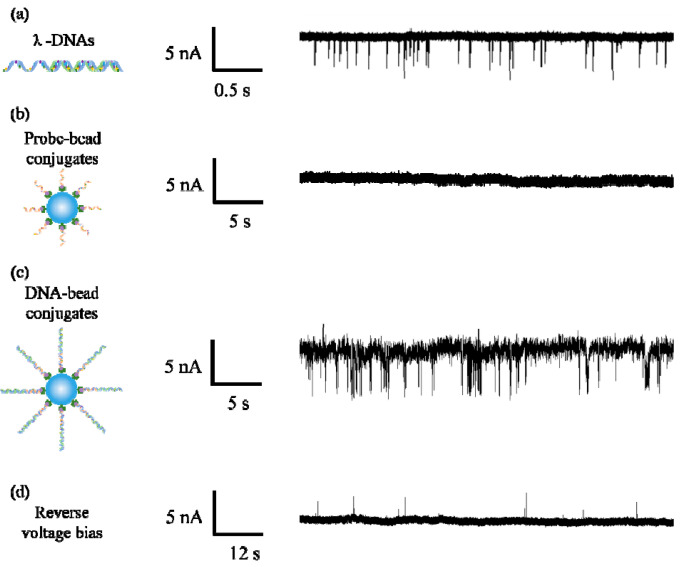
Ionic current traces for (**a**) λ-DNAs, (**b**) probe–bead conjugates and (**c**) DNA–bead conjugates; (**d**) ionic current trace measured under reversed the bias voltage.

We first detected λ-DNA molecules using a 35 nm diameter nanopore in 1M KCl solution under 800 mV bias voltage. As shown in Figure 3a, a number of signature pulse signals were generated in the trans-pore ionic current, which means free λ-DNA molecules can be driven through the pore by electrophoretic force. After finishing free the λ-DNA detection experiment, the chambers were washed by deionized water three times and the ionic current monitored for about 10 min to make sure no DNA remained in the chamber. Then a control experiment was performed on the microbeads with a probe attached. We did not observe any pulse signal during a 30-min measurement. On one hand, the bead is too large to enter the pore. On the other hand, for the small sized nanopore, electric field force hardly drags the beads to block the pore mouth. Therefore, for the DNA with mismatched sequence, the probe attached microbeads in the test sample will not generate pulse signals. 

When we detected DNA–bead conjugates by using the same pore, a large amount of pulse signals appeared in the current trace as shown in Figure 3c. We speculated that there are two possibilities which cause the appearance of pulse signals. The first one is that DNA entered in the pore, and then was pulled out of the pore due to the thermal motion of the microbead. The second one is that DNA passed through the pore after being released from the microbead. To explore the motion of DNA, we reversed the bias voltage to −800 mV after recording thousands of pulse signals. As shown in Figure 3d, several pulse signals appeared in ionic current, which means some released DNA molecules were recaptured into the pore from the trans side. It confirms DNA molecules successfully dissociated from the microbeads and transported through the pore, because DNA–bead conjugates were only added into the cis chamber. Furthermore, the link between λ-DNA and the biotin molecule is a covalent bond. In addition, streptavidin is also covalently bonded to the polystyrene microbead. The rupture force of the covalent bond is approximately 2–10 nN [45]. On the other hand, the streptavidin–biotin bond is a noncovalent bond, which is the weakest link in DNA–bead conjugates. Merlel et al. [46] indicated the breakage strength of the streptavidin–biotin bond ranges from 5 to 170 pN depending on the loading rate. The electrical force on a λ-DNA molecule in the nanopore can be calculated as *F* = *q*_eff_∆*V*/*a*, where *q*_eff_ is the effective charge of a DNA base pair, ∆*V* is the applied voltage across the nanopore, *a* refers to the distance between two base pairs [47]. In our experimental condition, the estimated electrical force on the DNA is about 192 pN at 800 mV, which is also larger than the streptavidin–biotin bond strength. 

**Figure 4:**
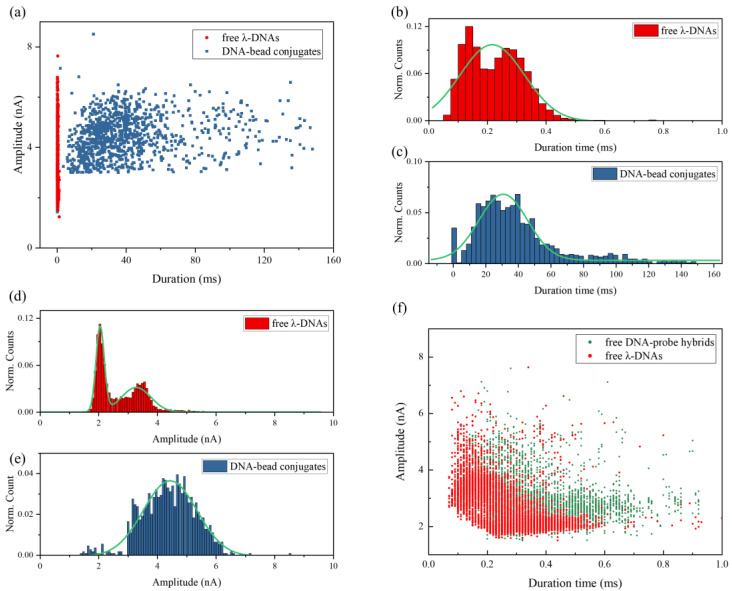
(**a**) Scatter plots of signal duration time versus amplitude for free λ-DNAs and DNA–bead conjugates; duration time distributions for (**b**) free λ-DNAs and (**c**) DNA–bead conjugates with Gaussian fits; amplitude distributions for (**d**) free λ-DNAs and (**e**) DNA–bead conjugates with Gaussian fits; (**f**) scatter plots of signal duration time versus amplitude for free λ-DNAs detected by using a 35 nm diameter nanopore and free DNA–probe hybrids detected by using a 25 nm diameter nanopore.

In order to further analyze the pulse signals generated by DNA–bead conjugates, we plotted the signal scatter diagram of duration time *t*_d_ versus current amplitude Δ*I* in Figure 4a. It is obvious that DNA–bead conjugates and free λ-DNA can be easily identified according to the duration time of pulse signals. As shown in Figure 4b, the mean duration time of free λ-DNA translocation events is about 0.22 ± 0.01 ms. While the mean duration time of signals for DNA–bead conjugates is 30.69 ± 0.72 ms (Figure 4c), which is more than two orders of magnitude longer than the free DNA translocation time. It can be mainly attributed to two factors. Firstly, there is a survival time of the streptavidin–biotin bond breakage, which depends on the loading rate [46]. Secondly, the breakage process will slow down the velocity of DNA molecules as well. 

Figure 4d,e present the current amplitude histograms with the fit of Gaussian distribution. There are two peaks in the Δ*I* histogram of free λ-DNA. It is well known that the pulse amplitude refers to the effective blocked area during DNA transportation through the pore. The first blockade current value peak is interpreted as a single DNA molecule in the nanopore in a linear configuration. In addition, the second peak corresponds to the translocation events of a folded DNA molecule or two parallel DNAs [48]. Interestingly, the amplitude of most pulse signals for DNA-bead conjugates is larger than the first peak value in Figure 4d. According to the recapture events shown in Figure 3d, we speculated that the signals for DNA–bead conjugates were generated by DNA–probe hybrids releasing from the microbead and passing through the pore. However, the amplitude of pulse signals in Figure 3d, which corresponds to the released DNA–probe hybrids recaptured by nanopore, is nearly same as the amplitude of pulse signals for free λ-DNA molecules in Figure 3a. It indicates that the biotinylated probe barely influences the amplitude of pulse signals. In addition, the biotin is a small molecule (MW 244.3), which will not lead to a large difference of characteristic size between the DNA–biotinylated probe hybrid and λ-DNA. To further exclude the influence of the biotinylated probe, we also detected free DNA–biotinylated probe hybrids by using a 25 nm diameter nanopore. As shown in Figure 4f, the amplitude of signals for free DNA–probe hybrids is only a little bit larger than that for free λ-DNA molecules, but still much smaller than that for DNA-bead conjugates. It confirms that the biotinylated probe is not the main reason for the large amplitude of pulse signals generated by DNA–bead conjugates. Furthermore, it should be noted that the slight enhancement of signal amplitude for the 25 nm diameter nanopore is due to the decrease in pore size [44]. For small sized pores, the pore resistance dominates the total resistance which is the sum of pore resistance and access resistance. So, the presence of DNA in the smaller sized pore has a relatively larger effect on the ionic current.

Kobayashi et al. [49] imaged DNA–streptavidin complex formation in solution by using a high-speed atomic force microscope. They observed that one streptavidin could bind up to three DNA strands, which leads to an extremely high local concentration of λ-DNAs on the surface of microbeads. According to our previous work [50], the probability of co-translocation events increases with the concentration of DNA molecules. We think the signals with such high amplitude were generated by multiple DNA molecules being transported through the pore simultaneously. 

Therefore, target DNA molecules, which are selectively bonded to the microbeads, can be easily identified by the ionic pulse signals. In addition, we also tried to use the probe containing three mismatched base pairs to detect λ-DNAs. In that case, λ-DNAs could not hybridize with noncomplementary probes, or bond to the microbeads. The amplitude and duration time of ionic pulse signals for the noncomplementary sample are almost the same as free λ-DNAs. It validates the specificity on our methodology.

## 4. Conclusions

We proposed a nanopore-based sensing platform assisted by microbeads to identify DNA molecules with a specific sequence. The target DNA molecules were successfully linked to the microbead through a streptavidin–biotin bond. Ionic pulse signals detected by nanopore exhibit totally different translocation behaviors between DNA molecules attached on microbeads and free DNA molecules. Experimental results verified that DNA molecules could be pulled away from the microbeads and driven through the pore by 800-mV bias voltage. This process takes 30.69 ± 0.72 ms, which is two orders of magnitude longer than free DNA translocation time. Furthermore, DNA molecules attached on the microbeads are more likely to enter into the pore simultaneously, due to the high local concentration. Therefore, according to the difference of pulse signal distribution, DNA-bead conjugates and free DNA molecules can be easily discriminated. The simplicity and sensitivity of the method indicates its alluring prospect in a variety of biomedical applications.
